# *Nitrososphaera viennensis* gen. nov., sp. nov., an aerobic and mesophilic, ammonia-oxidizing archaeon from soil and a member of the archaeal phylum *Thaumarchaeota*

**DOI:** 10.1099/ijs.0.063172-0

**Published:** 2014-08

**Authors:** Michaela Stieglmeier, Andreas Klingl, Ricardo J. E. Alves, Simon K.-M. R. Rittmann, Michael Melcher, Nikolaus Leisch, Christa Schleper

**Affiliations:** 1 University of Vienna, Department of Ecogenomics and Systems Biology, Archaea Biology and Ecogenomics Division, Althanstr. 14, 1090 Vienna, Austria; 2 Cell Biology and LOEWE Research Centre for Synthetic Microbiology (Synmikro), Philipps-University Marburg, Karl-von-Frisch-Str. 8, 35043 Marburg, Germany

## Abstract

A mesophilic, neutrophilic and aerobic, ammonia-oxidizing archaeon, strain EN76^T^, was isolated from garden soil in Vienna (Austria). Cells were irregular cocci with a diameter of 0.6–0.9 µm and possessed archaella and archaeal pili as cell appendages. Electron microscopy also indicated clearly discernible areas of high and low electron density, as well as tubule-like structures. Strain EN76^T^ had an S-layer with p3 symmetry, so far only reported for members of the *Sulfolobales*. Crenarchaeol was the major core lipid. The organism gained energy by oxidizing ammonia to nitrite aerobically, thereby fixing CO_2_, but growth depended on the addition of small amounts of organic acids. The optimal growth temperature was 42 °C and the optimal pH was 7.5, with ammonium and pyruvate concentrations of 2.6 and 1 mM, respectively. The genome of strain EN76^T^ had a DNA G+C content of 52.7 mol%. Phylogenetic analyses of 16S rRNA genes showed that strain EN76^T^ is affiliated with the recently proposed phylum *Thaumarchaeota*, sharing 85 % 16S rRNA gene sequence identity with the closest cultivated relative ‘*Candidatus* Nitrosopumilus maritimus’ SCM1, a marine ammonia-oxidizing archaeon, and a maximum of 81 % 16S rRNA gene sequence identity with members of the phyla *Crenarchaeota* and *Euryarchaeota* and any of the other recently proposed phyla (e.g. ‘*Korarchaeota*’ and ‘*Aigarchaeota*’). We propose the name *Nitrososphaera viennensis* gen. nov., sp. nov. to accommodate strain EN76^T^. The type strain of *Nitrososphaera viennensis* is strain EN76^T^ ( = DSM 26422^T^ = JMC 19564^T^)*.* Additionally, we propose the family *Nitrososphaeraceae* fam. nov., the order *Nitrososphaerales* ord. nov. and the class *Nitrososphaeria* classis nov.

## Introduction

Microbes are immensely diverse and abundant, and inhabit virtually all environments on Earth. However, most of this microbial diversity remains undescribed, given that many novel organisms are fastidious and their isolation and cultivation is time-consuming or even impossible (Rappé & Giovannoni, 2003). Cultivation-independent techniques have increased our knowledge of microbial diversity and metabolism tremendously (Lane *et al.*, 1985; Marcy *et al.*, 2007; Rinke *et al.*, 2013; Schmidt *et al.*, 1991; Stein *et al.*, 1996), and have led to the proposal of several new bacterial and archaeal phyla (e.g. Elkins *et al.*, 2008; Gordon & Giovannoni, 1996; Huber *et al.*, 2002; Hugenholtz *et al.*, 1998; Nunoura *et al.*, 2011; Rinke *et al.*, 2013). In addition, metabolic predictions based on meta-omic studies can also support the design of media and consequently facilitate the cultivation of uncharacterized microorganisms (Tyson & Banfield, 2005). When used in combination, microbial cultivation and metagenomics represent a powerful toolbox to describe newly discovered microbial physiologies, and to simultaneously assess their ecological distribution and impact. The discovery of ammonia-oxidizing archaea (AOA) represents a successful example of such an integrated approach. These archaea, originally called ‘mesophilic Crenarchaeota’, were first identified in marine samples (DeLong, 1992; DeLong *et al.*, 1994; Fuhrman *et al.*, 1992) and were later detected in many more environments (Jurgens *et al.*, 1997; Karner *et al.*, 2001; MacGregor *et al.*, 1997; Ochsenreiter *et al.*, 2003; Preston *et al.*, 1996; Schleper *et al.*, 1997; Takai *et al.*, 2001). The first evidence for their metabolism and potential ecological role was given by the discovery of genes encoding a putative ammonia mono-oxygenase, the key enzyme for ammonia oxidation, through metagenomics (Hallam *et al.*, 2006b; Treusch *et al.*, 2005; Venter *et al.*, 2004) and through the cultivation and isolation of the first AOA, ‘*Candidatus* Nitrosopumilus maritimus’ SCM1 (Könneke *et al.*, 2005). The widespread occurrence of potential archaeal ammonia oxidizers in the environment has been confirmed by a large number of qualitative and quantitative molecular surveys (Adair & Schwartz, 2008; Alves *et al.*, 2013; de la Torre *et al.*, 2008; Francis *et al.*, 2005; He *et al.*, 2007; Hershberger *et al.*, 1996; Leininger *et al.*, 2006; Pester *et al.*, 2012; Reigstad *et al.*, 2008; Shen *et al.*, 2008; Wuchter *et al.*, 2006; Zhang *et al.*, 2008). Based on phylogenetic analyses of concatenated ribosomal protein sequences and full-genome comparisons with the first genome sequence of the putative AOA ‘*Candidatus* Cenarchaeum symbiosum’ (Hallam *et al.*, 2006a), Brochier-Armanet *et al.* (2008) suggested that the AOA comprise a new archaeal phylum, the *Thaumarchaeota*, which was subsequently supported by Spang *et al.* (2010) upon the inclusion of two more genome sequences of members of the new phylum.

Due to the difficulty in cultivating and purifying members of the *Thaumarchaeota* in laboratory cultures, pure isolates are still rare (Könneke *et al.*, 2005; Tourna *et al.*, 2011) and insights into the physiology of AOA are confined to studies of two pure cultures (Könneke *et al.*, 2005; Martens-Habbena *et al.*, 2009; Schouten *et al.*, 2008) or are based on enrichment cultures (de la Torre *et al.*, 2008; Hatzenpichler *et al.*, 2008; Jung *et al.*, 2011; Lehtovirta-Morley *et al.*, 2011; Santoro & Casciotti, 2011). ‘*Candidatus* Nitrosopumilus maritimus’ SCM1 is a representative of the marine I.1a group, which is one of the distinct lineages formed within the phylum *Thaumarchaeota*. With the isolation of strain EN76^T^, the first pure culture from soil and group I.1b was obtained, which represents the second major lineage of the *Thaumarchaeota*. The isolation of strain EN76^T^ confirmed ammonia oxidation by thaumarchaeotes in soil and expanded the metabolic spectrum of AOA to include the utilization of urea and organic substrates (Tourna *et al.*, 2011). These findings demonstrate the importance of pure cultures to the investigation of growth requirements, and will also help in future cultivation and purification experiments of other members of the *Thaumarchaeota*.

The open ocean, marine sediments and soil contain large numbers of microbes, with approximately 1.2×10^29^, 2.9×10^29^ and 2.6×10^29^ cells, respectively (Kallmeyer *et al.*, 2012; Whitman *et al.*, 1998). Molecular surveys based on *amoA* or 16S rRNA gene sequences have shown that members of the *Thaumarchaeota* make up a large fraction of the microbial biomass in all three habitats (Bates *et al.*, 2011; Karner *et al.*, 2001; Leininger *et al.*, 2006). With up to 20 % of the picoplankton in the marine environment (Karner *et al.*, 2001), up to 80 % of the microbiota in certain horizons of marine sediments (Durbin & Teske, 2010; Jorgensen *et al.*, 2012) and up to 1 % of the total microbiota in soil (Bates *et al.*, 2011; Leininger *et al.*, 2006), the total abundance of thaumarchaeotes seems to be comparable to that of other highly abundant bacterial phyla, even to that of the ubiquitous Proteobacteria. Based on their large numbers in many environments and their ability to perform the first and rate-limiting step in nitrification, the oxidation of ammonia to nitrite, members of the *Thaumarchaeota* are now considered to play a major role in the global nitrogen cycle (Alves *et al.*, 2013; Di *et al.*, 2009; Erguder *et al.*, 2009; Gubry-Rangin *et al.*, 2010; Hatzenpichler, 2012; Jia & Conrad, 2009; Nicol & Schleper, 2006; Offre *et al.*, 2009; Prosser & Nicol, 2012; Schauss *et al.*, 2009; Schleper & Nicol, 2010; Stahl & de la Torre, 2012; Stieglmeier *et al.*, 2014a; Wuchter *et al.*, 2006; Xia *et al.*, 2011; Zhalnina *et al.*, 2012). Several factors have been suggested as drivers of environmental adaptation and niche selection of thaumarchaeotes, such as pH and ammonia concentration (reviewed by Prosser & Nicol, 2012). For example, ‘*Candidatus* Nitrosopumilus maritimus’ SCM1 and other marine members of the *Thaumarchaeota* have been shown to have a higher affinity to ammonia than cultivated ammonia-oxidizing bacteria (AOB), and all strains in pure culture or enrichment are adapted to low concentrations of ammonia (Horak *et al.*, 2013; Martens-Habbena *et al.*, 2009; Prosser & Nicol, 2012). However, thaumarchaeotes are widespread in diverse habitats, exposed to wide ranges of pH and ammonia concentration, and recent studies suggest that they almost certainly harbour a broader range of physiological and/or metabolic properties than is currently known (Alves *et al.*, 2013; Durbin & Teske, 2010; Jorgensen *et al.*, 2012; Muller *et al.*, 2010; Mussmann *et al.*, 2011). In order to understand their ecological impact, it is important to identify and characterize in detail the metabolic functions and physiological versatility of more representatives of these archaea.

Although AOA have been studied intensively in the last decade (reviewed by e.g. Nicol & Schleper, 2006; Stahl & de la Torre, 2012; Stieglmeier *et al.*, 2014a), there are presently no validly published names of species, genera or higher ranks within the phylum *Thaumarchaeota*. Here, we extend the original characterization of strain EN76^T^ (Tourna *et al.*, 2011), with a special focus on its ultrastructure and growth improvements, and formally propose the species *Nitrososphaera viennensis* sp. nov. and propose to assign this species as the type species of the genus *Nitrososphaera* gen. nov. The genus *Nitrososphaera* is the type of the family *Nitrososphaeraceae* fam. nov. and the order *Nitrosophaerales* ord. nov., which is in turn the type of the class *Nitrososphaeria* classis nov.

## Methods

### 

#### Sample source and culture conditions.

Sample source and isolation strategy for strain EN76^T^ were described previously by Tourna *et al.* (2011). In brief, strain EN76^T^ was isolated from Viennese garden soil (48° 13′ 48.72″ N 16° 21′ 28.93″ E) by transferring 5 g soil into 50 ml freshwater medium (FWM) containing (l^−1^) 1 g NaCl, 0.4 g MgCl_2_ . 6H_2_O, 0.1 g CaCl_2_ . 2H_2_O, 0.2 g KH_2_PO_4_, 0.5 g KCl, 1 ml trace element mixture, 1 ml vitamin solution and 7.5 µM ferric sodium EDTA, 0.5 mM NH_4_Cl as energy source and 2 mM NaHCO_3_ as carbon source. Additionally 0.1 mM NaNO_2_ was supplied to the cultures. The medium was adjusted to pH 7.5 and cultures were incubated at 37 °C in the dark without shaking. Supplementation with antibiotics (carbenicillin, streptomycin) and filtration of the cultures (0.45 µm pore size) were applied to reduce bacterial and fungal contaminants. Growth was followed by measuring ammonium consumption and nitrite production photometrically. Additionally, light microscopy and quantitative PCR were used as described previously (Tourna *et al.*, 2011).

The purified strain EN76^T^ was routinely cultured in 20 ml FWM in sterile 30 ml polystyrene screw-capped containers (VWR; catalogue no. 216-2637) supplemented with 1 mM NH_4_Cl, 2 mM NaHCO_3_, 0.1 mM sodium pyruvate and 100 µg antibiotics ml^−1^ (kanamycin, streptomycin or ofloxacin in MilliQ water). The medium was buffered with HEPES/NaOH to pH 7.5. Larger volumes were cultured in glass bottles and shaken (150 r.p.m.) in darkness.

#### Physiological characterization and multivariate optimization of growth conditions.

To determine optimal growth parameters as well as the effect of different nitrogen and carbon substrates on the growth of EN76^T^, the strain was cultivated in closed batch in 20 ml FWM containing 100 µg antibiotics ml^−1^ as described above. Substrates were dissolved in MilliQ water and sterile filtered (0.2 µm) before usage. Table 1 gives an overview of tested substrates.

**Table 1.** Effect of substrates on growth of strain EN76^T^ The effect of various substrates on growth of EN76^T^ was evaluated compared with a chemolithoautotrophic culture supplemented with 1 mM ammonium and 2 mM bicarbonate (default culture). Growth is scored as: ++, positive effect; +, similar to default culture; +/−, negative effect; −, total inhibition of growth. All incubations were performed at least in duplicate. The table is an extended version of Table S1 of Tourna *et al.* (2011). The default ammonium concentration added to cultures was 1 mM (if not stated otherwise) and the default pyruvate concentration added to cultures was 0.5 mM (if not stated otherwise).

**Substrate**	**Substrate added**		**Growth**
	**Ammonium**	**Pyruvate**	
**Carbon compounds**			
Bicarbonate >2 mM (up to 10 mM)	Yes	No	+
Organic acids (TCA cycle)			
Acetate (0.05–2 mM)	Yes	No	+
Citrate, succinate, fumarate, malate (0.1–0.5 mM)	Yes	No	+
Pyruvate (0.05–10 mM)	Yes	Yes	++
Pyruvate (0.5 mM)	No	Yes	−
Oxaloacetate (0.5 mM)	Yes	No	++
α-Ketoglutarate (0.5 mM)	Yes	No	++
Glyoxylate (0.5 mM)	Yes	No	++
Sugars and sugar alcohols			
Glucose, fructose, lactose, arabinose, sucrose, galactose, mannose (0.5–1 mM)	Yes	No	+
Ribose (0.5 mM)	Yes	No	−
Glycerol (0.1 %, v/v)	Yes	No	−
Complex organic compounds			
Peptone, yeast extract (0.05 %, w/v)	Yes	No	−
**Nitrogen compounds**			
Ammonium			
0.5–3 mM	Yes	No	+
0.5–15 mM	Yes	Yes	++
Urea			
0.5–1 mM	No	No	+
0.5 mM	No	Yes	++
Amino acids			
l-Alanine, d-alanine, l-glutamine, l-aspartic acid (0.1 g l^−1^)	Yes	No	−
l-Glutamic acid, d-glutamic acid (0.1 g l^−1^)	Yes	No	+
Amino acid mixture (1 mM), Casamino acids (0.05 %, w/v)	Yes	No	−
Amines			
Trimethylamine, ethanolamine (1 mM)	No	No	+/−
Methanolamine (1 mM)	No	No	−
Methylamine (0.5 mM)	Yes	No	+/−
Nitrate (1 mM)	No	Yes	−
Taurine			
0.25–0.5 mM	Yes	No	+
0.25–0.5 mM	Yes	Yes	++
Nucleobases			
Pyrimidine, purine (0.1–1 mM)	Yes	No	+/−
Cytidine (0.1–1 mM)	Yes	No	+

**Fig. 1.**
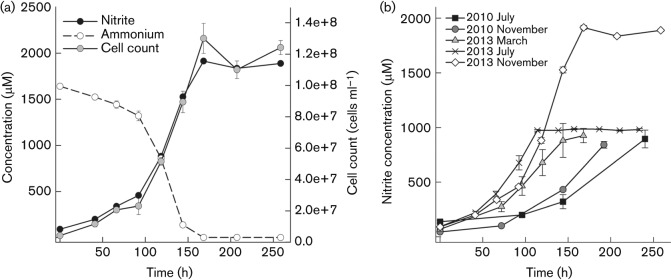
(a) Growth curve of a culture of strain EN76^T^ grown at 42 °C with 2 mM NH_4_Cl and 1 mM pyruvate. Cell counts, ammonium consumption and nitrite production were used to follow growth. Data represent mean values of triplicate cultures with standard deviations plotted (sometimes smaller than symbols). (b) Acceleration of growth of strain EN76^T^ since purification of the strain in 2010 (Tourna *et al.*, 2011). The cultivation conditions were as follows: July 2010 and November 2010, 37 °C, 1 mM NH_4_Cl, 1 mM pyruvate; March 2013, 37 °C, 1 mM NH_4_Cl, 0.1 mM pyruvate; July 2013, 42 °C, 1 mM NH_4_Cl, 0.8 mM pyruvate; November 2013, 42 °C, 2 mM NH_4_Cl, 1 mM pyruvate. Nitrite production was used to follow growth. Data represent mean values of replicated cultures (three to five replicates) with standard deviations plotted (sometimes smaller than symbols). Data points previously published in Fig. 3(b) of Tourna *et al.* (2011) (i.e. July 2010) were included in the figure.

In order to investigate optimal growth conditions for EN76^T^, a design of experiments (DoE) strategy was applied, using the factors temperature, pyruvate concentration and ammonium concentration. Based on our preliminary knowledge of the strain’s growth requirements (Tourna *et al.*, 2011), the range for each factor (design space) was set as follows: 37–47 °C, 0.1–1.5 mM sodium pyruvate and 1–4 mM NH_4_Cl. As nitrite production was shown to follow biomass production (Tourna *et al.*, 2011), it was used to calculate the growth rate (μ) and maximum growth rate (μ_max_), which were eventually used to develop the model (Design-Expert 8 software; Stat-Ease Inc.). Experiments were conducted in triplicate, except for the centre points of the initial two-level factorial screening design, which were set up in fivefold replicates. The two-level factorial design was applied in order to screen the design space rapidly. Due to a low model significance of data obtained from the initial two-level factorial screening design space, an augmented matrix was used in order to account for putative interactions of individual factors. Thus, the two-level factorial design space was extended by using a face-centred augmented matrix. Eventually, data points of all experiments (*n* = 51) were used to establish a response surface model (RSM). Data were analysed with the software Design-Expert 8. ANOVA, based on a stepwise regression elimination procedure, was used to develop the model. The desirability approach, as described elsewhere (Derringer & Suich, 1980), was used to maximize μ or μ_max_ (variable) based on variation of quantitative factors, here c_(ammonium)_, c_(pyruvate)_ and temperature (within the design space). A score is given to each quantitative factor setting that can be used to maximize the variable. In this approach, desirability between 0 and 1 (corresponding to 0–100 %) can be assigned to a variable for optimization; factors identified as being outside a certain desirability function will not be considered for model generation. To verify the calculated optimal growth conditions identified by the established RSM model design space, one additional growth experiment (fivefold-replicated closed-batch cultures) was performed (Fig. 1a).

For the cultivation of strain EN76^T^ under reduced oxygen concentrations, serum bottles were sealed with rubber stoppers. Therefore, the effect of black butyl (Glasgerätebau Ochs), grey butyl (Sigma Aldrich), blue butyl (Dunn Labortechnik), red isoprene (Sigma Aldrich), grey natural (Carl Roth) and grey–blue natural (VWR) rubber stoppers on the growth of strain EN76^T^ was tested (Table S1, available in the online Supplementary Material).

#### Microscopy.

For negative staining, cells were fixed with 2.5 % glutaraldehyde in 1× PBS, transferred to carbon-coated copper grids (200 mesh) and stained with 0.5 % uranyl acetate for 2 min as described previously (Tourna *et al.*, 2011). Images were recorded with a megaview III camera (SIS) attached to a Philips EM 208 transmission electron microscope (FEI) operated at 70 keV.

For scanning electron microscopy (SEM), poly-l-lysine-coated glass slides were added to the culture from early to late exponential phase. Attached cells were fixed for 2 h at room temperature with 2 % glutaraldehyde and 2 % formaldehyde in 0.06 × PHEM buffer (buffer based on PIPES, HEPES, EGTA and MgCl_2_; J. Montanaro and N. Leisch, unpublished). The cells were post-fixed with 1 % osmium tetroxide for 2 h at room temperature followed by dehydration in a graded ethanol series. After immersion in pure acetone, the slides were critical-point-dried with a CPD 300 unit (Leica). The slides were then mounted on stubs, gold-coated with an AGAR B7340 sputter-coater and imaged using an XL20 instrument (Philips) running the Microscope control program (version 7.00; FEI).

For the preparation of ultrathin sections, cells were grown in 1 l FWM supplemented with 3 mM NH_4_Cl and 0.15 mM sodium pyruvate until the late exponential growth phase. Cells were harvested by centrifugation and fixed as mentioned above for SEM preparation. After fixation, cells were washed twice in 100 mM PHEM buffer and covered with 1 % agar. Cells were post-fixed for 1 h in osmium tetroxide (1 %), washed three times (1×PHEM; J. Montanaro and N. Leisch, unpublished), dehydrated in a graded ethanol series and embedded in resin with acetonitrile as solvent. Polymerization was achieved by incubating the resin blocks for 1 h at 40 °C and for 48–72 h at 60 °C (Leisch *et al.*, 2011). An Ultracut S (Leica) was used to produce ultrathin sections (70 nm), which were then transferred to copper grids (300 mesh). They were post-stained with uranyl acetate and lead citrate before visualization on a Zeiss 902 instrument equipped with an Olympus SharpEye camera at an accelerating voltage of 80 keV and a Libra120 instrument (Carl Zeiss) equipped with a slow-scan CCD camera (Tröndle) at an accelerating voltage of 120 keV. Images were post-processed with Adobe Photoshop CS5.

To determine the general morphology and properties of the potential S-layer protein of EN76^T^, negative staining of purified S-layer sheets as well as freeze-fracturing/freeze-etching was performed. If not otherwise mentioned, freeze-etching was carried out as described previously (Klingl *et al.*, 2011; Rachel *et al.*, 2010). The purification of S-layer proteins was done by breaking the cells by sonication, differential centrifugation and extraction of lipids, using a MES buffer system (Klingl, 2011; Veith *et al.*, 2009). Subsequent negative staining of S-layer sheets for transmission electron microscopy (TEM) was performed as described previously (Rachel *et al.*, 2010). For the investigation of freeze-etching replicas and purified S-layer proteins, a JEOL JEM 2100 TEM, equipped with a fast scan 2k×2k CCD camera F214 (TVIPS), was used at an accelerating voltage of 120 kV. Image analyses, correlation averaging and determination of S-layer symmetry and lattice constants of negatively stained S-layer proteins as well as freeze-etching replicas was performed with the animetra crystals software package (release 1.1; Animetra).

#### Lipid analyses.

Intact polar lipids and glycerol dibiphytanyl glycerol tetraether (GDGT) were analysed previously and described by Sinninghe Damsté *et al.* (2012).

#### DNA isolation and phylogenetic analyses.

Isolation of DNA from the enrichment culture of strain EN76^T^ and 454 pyrosequencing on a 454/FLX-Titanium sequencer (Roche) have been described in detail previously (Tourna *et al.*, 2011). The genome sequence was assembled and annotated on the MicroScope platform (Vallenet *et al.*, 2006, 2009). The 16S rRNA, *amoA* and *amoB* gene sequences of EN76^T^ have been deposited previously in GenBank under accession numbers FR773157, FR773159 and FR773160, respectively (Tourna *et al.*, 2011). Phylogeny reconstruction of archaeal 16S rRNA genes was based on the alignment of 1202 bp gene fragments with four independent methods: clustal w (Thompson *et al.*, 1994), muscle (Edgar, 2004), mafft (Katoh *et al.*, 2005; Katoh & Toh, 2010), and T-Coffee (Di Tommaso *et al.*, 2011). The final consensus multiple sequence alignment of the four methods was calculated with MergeAlign (Collingridge & Kelly, 2012). After manual curation, hypervariable positions that could not be aligned unambiguously were excluded. Maximum-likelihood phylogenetic trees and bootstrap support values were calculated with RaxML VI-HPC (Stamatakis, 2006; Stamatakis *et al.*, 2008) based on the GTR model with invariable sites and gamma site rate variation (GTR+I+G). clustal w, muscle, mafft and RaxML analyses were performed through the CIPRES Science Gateway version 3.3 (Miller *et al.*, 2010).

#### Storage.

Cells of strain EN76^T^ were harvested, suspended in 40 % (v/v) glycerol and stored at −80 °C. Growth could be restored after preservation for 12 months by carefully thawing cells on ice and removing the glycerol by centrifugation of cells prior to inoculation into fresh medium.

## Results and Discussion

### Metabolism

Strain EN76^T^ is a mesophilic and neutrophilic organism, growing at 28–47 °C and pH 6–8.5 (Tourna *et al.*, 2011). It produces energy by oxidizing ammonia aerobically to nitrite (Fig. 1a). Strain EN76^T^ grows equally well on urea as an energy source, with production of about 2 mmol nitrite per mol urea (Tourna *et al.*, 2011). In contrast to ‘*Candidatus* Nitrosopumilus maritimus’ SCM1 (group I.1a), strain EN76^T^ and its close relative ‘*Candidatus* Nitrososphaera gargensis’ Ga9.2 (both associated with group I.1b) possess genes encoding urease and urea transporters (Spang *et al.*, 2012; Stieglmeier *et al.*, 2014a; Tourna *et al.*, 2011; Walker *et al.*, 2010) and, accordingly, growth on urea has also been demonstrated in enrichment cultures of ‘*Candidatus* Nitrososphaera gargensis’ Ga9.2 (Spang *et al.*, 2012). Genes for urea utilization were recently also found in metagenomic analyses of marine group I thaumarchaeotes from Arctic and meso-/bathypelagic waters and marine sediments, indicating that urea is a more widespread energy (and perhaps also carbon) source for AOA (Alonso-Sáez *et al.*, 2012; Park *et al.*, 2012) than was previously appreciated. The NO scavenger carboxy-PTIO [2-(4-carboxyphenyl)-4,4,5,5-tetramethylimidazoline-1-oxyl-3-oxide] (Amano & Noda, 1995) has been shown to inhibit growth and nitrite production of both strain EN76^T^ (Shen *et al.*, 2013) and ‘*Candidatus* Nitrosopumilus maritimus’ SCM1 (Yan *et al.*, 2012), indicating an important role for NO in their energy metabolism. In addition, hydroxylamine is probably an intermediate during the oxidation of ammonia to nitrite, as shown for AOB [Arp & Stein (2003) and references therein; Hooper & Terry (1979)], and recently also shown for the marine AOA ‘*Candidatus* Nitrosopumilus maritimus’ SCM1 (Vajrala *et al.*, 2013). Strain EN76^T^ tolerated ammonium concentrations up to 15 mM and nitrite concentrations up to 10 mM, as reported previously (Tourna *et al.*, 2011). Thus, EN76^T^ is less sensitive to high ammonium and nitrite concentrations than, for example, its close relative ‘*Candidatus* Nitrososphaera gargensis’ Ga9.2 and the marine strain ‘*Candidatus* Nitrosopumilus maritimus’ SCM1 (Hatzenpichler *et al.*, 2008; Könneke *et al.*, 2005). The strain produces nitrous oxide (N_2_O) in amounts comparable to those of AOB under oxic conditions (4.6±0.6 amol N_2_O cell^−1^ h^−1^). However, in contrast to AOB, N_2_O production does not increase under reduced oxygen levels, and might occur via a hybrid formation mechanism (Stieglmeier *et al.*, 2014b).

Strain EN76^T^ is a mixotrophic organism that requires organic acids (e.g. pyruvate, oxaloacetate, α-ketoglutarate or glyoxylate) to stimulate growth (Table 1; Tourna *et al.*, 2011). Other organic substrates, such as sugars or amines, did not have a positive effect on growth of EN76^T^ (Table 1). Growth stimulation by organic acids has recently also been reported for the marine strain ‘*Candidatus* Nitrosopumilus maritimus’ SCM1 (Stahl & de la Torre, 2012; Urakawa *et al.*, 2011). Comparative genomic analyses of strain EN76^T^, ‘*Candidatus* Nitrosopumilus maritimus’ SCM1 (Walker *et al.*, 2010) and ‘*Candidatus* Nitrososphaera gargensis’ Ga9.2 (Spang *et al.*, 2012) revealed the presence of genes encoding transporters for amino acids, sulfonates (e.g. taurine) and glycerol in all three strains (P. Offre, M. Kerou, A. Spang and C. Schleper, unpublished). In addition, EN76^T^ encodes putative nucleobase transporters. Therefore, different amino acids, taurine, glycerol and nucleobases were tested in various concentrations as possible substrates for EN76^T^ (Table 1). However, none of the above-mentioned compounds had a positive effect on growth under the conditions tested. Instead, glycerol, several amino acids and nucleobases inhibited growth.

Strain EN76^T^ was initially described to grow optimally in FWM supplemented with 1 mM pyruvate and 1 mM NH_4_Cl at 37 °C and pH 7.5 (Tourna *et al.*, 2011), with a doubling time of approximately 45 h (based on cell counts). However, the generation time of strain EN76^T^ decreased progressively from 45 to 27.5 h during continuous cultivation and growth optimizations over 3 years (Fig. 1b). In order to reassess the optimal growth conditions and possible interactions between the three known factors that influence growth rate (i.e. temperature and ammonium and pyruvate concentrations) comprehensively, we used a DoE screening and optimization strategy (Box & Draper, 1987; Box & Lucas, 1959; Rittmann & Herwig, 2012). As an example for the output of those experiments, the RSM shown in Fig. S1 illustrates the effect of temperature and pyruvate concentration on the maximum specific growth rate (μ_max_) at a constant ammonium concentration of 2.5 mM. Based on the DoE data and the calculated model obtained after varying temperature, ammonium and pyruvate concentrations, we predicted the optimal growth conditions of EN76^T^ (see Table S2). The highest maximum specific growth rate (μ_max_ 0.024 h^−1^) should be reached at 41.83 °C, 1.05 mM pyruvate and 2.59 mM NH_4_Cl (with a desirability of 84 %). This corresponds to a generation time of 29.0 h. In order to verify μ_max_ and the generation time predicted by the RSM experimentally, we grew the strain at 42 °C, 1 mM pyruvate and 2 mM NH_4_Cl and obtained a maximum specific growth rate (μ_max_) of 0.023 h^−1^ and a generation time of 30.1±0.6 h (based on nitrite production). Similar values for growth rate and generation time were obtained using cell counts of EN76^T^ for the calculation (μ_max_ 0.026 h^−1^; generation time 27.5±6.5 h). These experimentally determined values are close to the generation time (29.2 h) and a maximum specific growth rate (μ_max_ 0.024 h^−1^) predicted by the model equation under the tested conditions (Table S2). The model equation was additionally verified by recalculating the generation time reported by Tourna *et al.* (2011). The generation time based on the previously used growth conditions was calculated as 42.0±2.7 h, which is close to the initial experimental determination of 45 h (Tourna *et al.*, 2011). Enrichment cultures of the closely related strain ‘*Candidatus* Nitrososphaera gargensis’ Ga9.2 have been reported to grow at 46 °C with an ammonium concentration of 1 mM (Hatzenpichler *et al.*, 2008).

Given that thaumarchaeotes have been shown to be light-sensitive (French *et al.*, 2012; Merbt *et al.*, 2012), strain EN76^T^ was incubated in the dark. When cultivated in larger volumes (>100 ml), cultures were shaken at 150 r.p.m. Although the strain grows aerobically [21 % (v/v) O_2_ in the gas phase], it can grow at oxygen concentrations as low as 3 % (v/v) O_2_ in the gas phase (Stieglmeier *et al.*, 2014b).

Tests of various rubber stoppers indicated that growth of EN76^T^ was inhibited completely by black butyl and red isoprene rubber stoppers, although it tolerated grey and blue butyl rubber stoppers, as well as grey natural rubber stoppers (Table S1). Inhibition of the activity of methanotrophic bacteria by black butyl rubber stoppers has been reported previously (Ettwig *et al.*, 2009).

EN76^T^ is not affected by water-soluble antibiotics like kanamycin, streptomycin, carbenicillin, ampicillin (Tourna *et al.*, 2011) and ofloxacin, but is inhibited by antibiotics that are soluble in ethanol or DMSO (e.g. chloramphenicol). Recently, the effects of nitrification inhibitors (e.g. nitrapyrin, allylthiourea and dicyandiamide) and the antibiotic sulfathiazole, which are commonly used in agriculture and livestock production, on the AOA strain EN76^T^ and a strain of the ammonia-oxidizing bacterium *Nitrosospira multiformis* have been tested (Shen *et al.*, 2013). Nitrapyrin had a stronger inhibitory effect on EN76^T^ compared with the bacterium, whereas dicyandiamide, the copper chelators allylthiourea and amidinothiourea and the antibiotic sulfathiazole had a weaker inhibitory effect on EN76^T^ (Shen *et al.*, 2013).

### Morphology

**Fig. 2.**
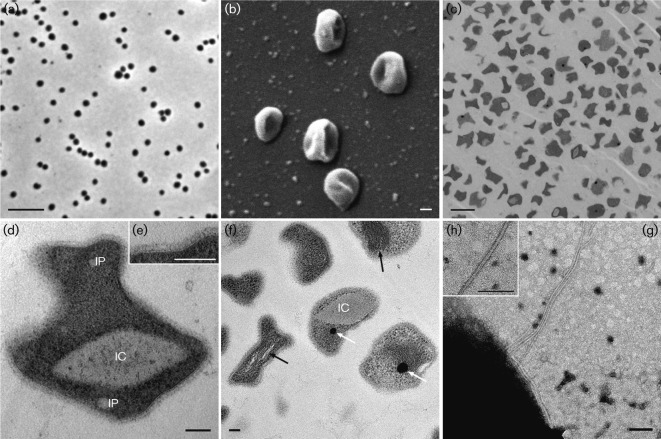
Ultrastructure of cells of strain EN76^T^. (a) Phase-contrast image; bar, 5 µm. (b) Scanning electron micrograph of several cells depicting the irregular coccoid shape; bar, 100 nm. (c–f) TEM images of ultrathin sections of chemically fixed cells of strain EN76^T^. (c) Overview displaying the irregular cell shape; bar, 1 µm. (d) Magnified cell showing intracellular features including a clearly discernible area [potential intracellular compartment (IC)] and incorporations (IP). The inset (e) illustrates the cell membrane, pseudo-periplasm and S-layer at higher magnification; bars, 100 nm. (f) Potential intracellular compartment (IC), tubule-like structures (white arrows) and electron-dense particles (black arrows) are highlighted; bar, 100 nm. (g, h) Transmission electron micrographs of a cell with an archaellum; inset (h) shows the magnified archaellum. Bars, 100 nm.

The irregular coccoid cells of strain EN76^T^ had a diameter of 0.78±0.13 µm (*n* = 16) and usually occurred as single cells, although clusters comprising several cells were sometimes observed. Cells were motile and possess archaella (archaeal flagella that are homologous to type IV pili; Jarrell & Albers, 2012) with a diameter of 12.0±1.8 nm (Fig. 2g, h). Genes encoding *Crenarchaeota*-like type-2 flagellins and *Euryarchaeota*-like chemotaxis proteins (Fig. S2) were found in the genome of EN76^T^, supporting the conclusion that EN76^T^ is probably motile. *Crenarchaeota*-like type-2 *fla* gene clusters have been found in ‘*Candidatus* Nitrososphaera gargensis’ Ga9.2 and the group I.1a-related strain ‘*Candidatus* Nitrosoarchaeum limnia’ SFB1, but not in the genome of ‘*Candidatus* Nitrosopumilus maritimus’ SCM1 (Blainey *et al.*, 2011; Spang *et al.*, 2012; Walker *et al.*, 2010). In addition, pili with a diameter of 6.4±1.3 nm were observed (not shown). The diameters of both appendages are within the size ranges described for other archaea (Klingl *et al.*, 2013).

In order to investigate the ultrastructure of EN76^T^, ultrathin sections of chemically fixed and embedded cells were prepared (Fig. 2c–f). Cells often contained one or two electron-dense particles with a size of 87±15 nm (Fig. 2f). Similar electron-dense particles present in anaerobic ammonium-oxidizing (anammox) bacteria have been shown to be enriched in iron and were proposed to constitute bacterioferritins, which are known as iron storage proteins (Andrews *et al.*, 2003). Furthermore, the genome of EN76^T^ carries genes for proteins belonging to the ferritin/Dps domain proteins (Nvie_002890, Nvie_017250, Nvie_020180, Nvie_028750, Nvie_029390), which have also been identified in genomes of other members of the *Thaumarchaeota* (Spang *et al.*, 2012). Dps proteins are known to protect cells against oxidative stress by binding iron (Haikarainen & Papageorgiou, 2010). These findings suggest that the electron-dense particles present in strain EN76^T^ may have a role in iron storage and cellular protection. Tubule-like structures were also identified in cells of strain EN76^T^ (Fig. 2f), and may have a cytoskeletal function, as proposed previously for the hexagonal long tubule-like structures found inside the anammoxosome of anammox bacteria (Lindsay *et al.*, 2001; van Niftrik & Jetten, 2012). Although genomes of members of the *Thaumarchaeota* encode an FtsZ homologue, it is unlikely that the tubule-like structures are formed by this protein, because the marine strain ‘*Candidatus* Nitrosopumilus maritimus’ SCM1 was shown to recruit the Cdv mechanism primarily during cell division (Pelve *et al.*, 2011). Small inclusions of less electron-dense material (compared with the cytoplasm) were observed in cells of strain EN76^T^ (Fig. 2d), which might be polyphosphate or glycogen storage granules, for example (Klingl *et al.*, 2013). Strain EN76^T^ possesses a clearly discernible area within the cytoplasm (Fig. 2d, e). So far, we could not identify a lipid or proteinaceous layer surrounding this area, which is characteristic for intracellular microcompartments found in bacteria. Large intracellular compartments, such as the carboxysome and anammoxosome, have been described in several bacteria (Erbilgin *et al.*, 2014; Shively *et al.*, 1973; van Niftrik *et al.*, 2004). However, compartmentalization has been reported in only few archaea, e.g. a two-membrane system in the crenarchaeotal genus *Ignicoccus* (Huber *et al.*, 2000) and in the euryarchaeote *Methanomassiliicoccus luminyensis* (Dridi *et al.*, 2012). Larger intracellular particles like polyhydroxyalkanoate granules have been found in halophilic archaea (Fernandez-Castillo *et al.*, 1986). Raman spectroscopy analyses have indeed shown that strain EN76^T^ also synthesizes polyhydroxyalkanoates (Spang *et al.*, 2012). Further analyses will be necessary to show whether this region is separated by a membrane or proteinaceous layer from the cytoplasm, and to identify the function of this potential intracellular microcompartment.

**Fig. 3.**
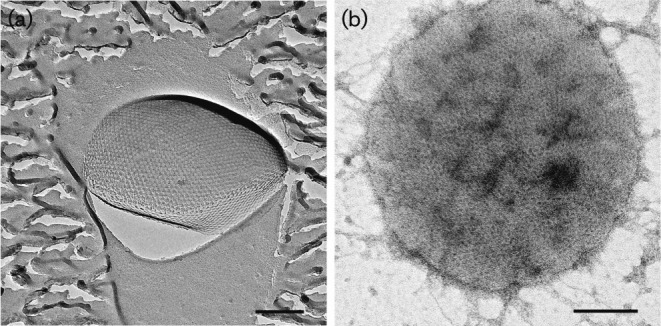
Electron micrographs of a freeze-etching replica (a) and a negatively stained purified S-layer sheet (b) of a cell of strain EN76^T^. Bars, 200 nm.

**Fig. 4.**
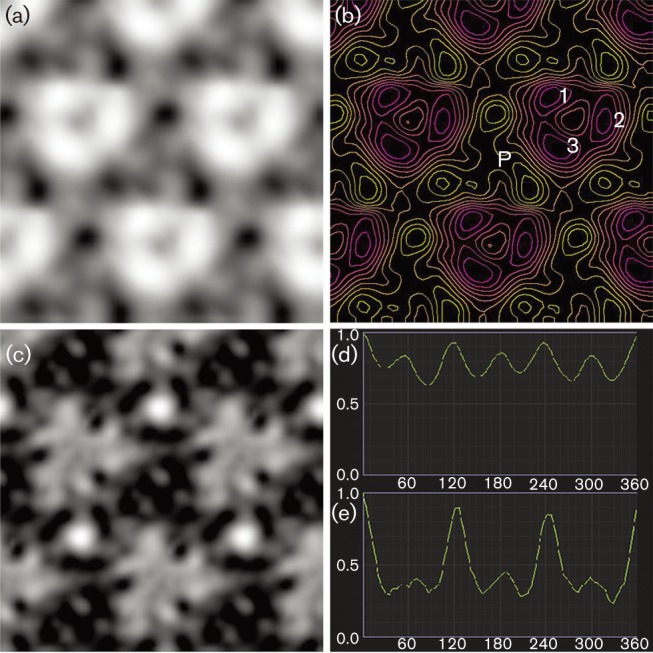
Determination of S-layer symmetry of EN76^T^. (a) Correlation averaging of the freeze-etched S-layer from Fig. 3(a), showing the protein subunits (white areas) and pores (grey and black areas). (b) Relief reconstruction of the averaged image from (a). The crystal unit cell probably consists of a trimer of protein-trimers, of which one is indicated (1–3). Elevated areas are labelled violet and red and depths are labelled yellow, revealing a triangular cavity or pore (P). (c) Correlation averaging of the negatively stained S-layer from Fig. 3(b). Similar to (a), the proteins are represented by white and light-grey areas and uranyl acetate-filled cavities by dark-grey and black areas. (d) and (e) show the determination of S-layer symmetry of the unit cells in (a) and (c), respectively. The images were tilted by increments of 5° and the correlation with the original, untilted image (set as 1) is plotted against the tilting angle.

S-layer proteins are one, or even the only, cell-wall component of several archaea and bacteria (Rachel *et al.*, 1997). The S-layer usually consists of a single type of protein arranged in a regular lattice pattern, probably driven by entropic processes (Eichler, 2003; Sleytr *et al.*, 2001, 2007). Depending on the arrangement, these pseudocrystalline areas depict highly ordered opaque p1- or p2-symmetry, square p4-symmetry or sixfold p3- or p6-symmetry. TEM analyses of both freeze-etching replicas (Fig. 3a) and negatively stained S-layer sheets (Fig. 3b) of cells of strain EN76^T^ showed a regular pattern of a two-dimensional protein crystal with 6-fold symmetry. By further analysing the images with animetra crystals, distinction between p3- and p6-symmetry could be achieved. Correlation averaging of electron micrographs of both freeze-etched cells (Fig. 4a) and purified S-layer sheets (Fig. 4c) revealed an unexpected p3-symmetry of the S-layer protein. The relief illustration of the image in Fig. 4(a), shown in Fig. 4(b), revealed that the unit cell of the S-layer consists of a trimer of protein trimers. Additionally, the triangular shape of the pores between the protein trimers can be seen, which could function as a molecular sieve, separating the surrounding medium from the pseudo-periplasm, located between the S-layer and the cytoplasmic membrane (reviewed by Sleytr *et al.*, 1993). Determination of the lattice constants after correlation averaging yielded slightly differing values for the two preparation methods, with 21.1 nm for freeze-etching and 20.2 nm for negative staining. This could be caused by the preparation itself, by calculation errors or by the initial choice of the reference area for correlation averaging. The range of lattice values obtained here is, nevertheless, in the same range as those for other p3-symmetry S-layers reported for members of the *Sulfolobales*, which are all around 21 nm (König *et al.*, 2007; Veith *et al.*, 2009). Up to now, p3-symmetry was thought to be unique to the order *Sulfolobales*, given that all investigated species from this group had this symmetry and a consistent lattice value (Baumeister & Lembcke, 1992; Deatherage *et al.*, 1983; Grogan, 1996; Klingl *et al.*, 2013; König *et al.*, 2007; Lembcke *et al.*, 1991, 1993; Prüschenk & Baumeister, 1987; Prüschenk *et al.*, 1987; Taylor *et al.*, 1982; Veith *et al.*, 2009). Thus, the occurrence of p3-symmetry in the thaumarchaeote EN76^T^ excludes this characteristic as a taxonomic marker for the order *Sulfolobales* (Klingl *et al.*, 2011; König *et al.*, 2007).

As described previously (Sinninghe Damsté *et al.*, 2012), the intact polar lipids of cells of strain EN76^T^ consist of GDGTs bound to the polar head groups monohexose, dihexose, trihexose, phosphohexose or hexose-phosphohexose. Crenarchaeol and its regioisomer, both GDGTs with one cyclohexane and four cyclopentane rings (Sinninghe Damsté *et al.*, 2002), were the major core lipids (80 %; Sinninghe Damsté *et al.*, 2012). So far, crenarchaeol has been found exclusively in members of the *Thaumarchaeota*.

### Phylogenetic analyses

**Fig. 5.**
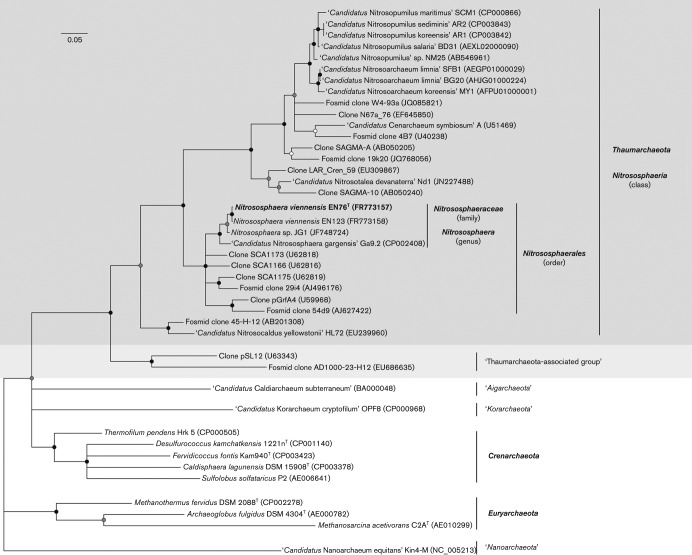
Maximum-likelihood 16S rRNA gene phylogeny of the *Thaumarchaeota* and representative strains of the *Crenarchaeota*, *Euryarchaeota* and other proposed archaeal phyla. The tree depicts *Nitrososphaera viennensis* EN76^T^ (bold), the marine pure culture ‘*Candidatus* Nitrosopumilus maritimus’ SCM1, organisms from laboratory or natural enrichment cultures (labelled *Candidatus*) and a selection of environmental sequences representing major uncultured lineages. Proposed phyla and uncharacterized archaeal lineages are placed in quotes. Phylogeny reconstruction was based on 1202-bp 16S rRNA gene fragments and calculated with RaxML VI-HPC using the GTR+I+G model. Bootstrap support values (1000 replicates) are indicated by circles: filled, ≥90 %; shaded, ≥80 % but <90 %; open, ≥70 % but <80 %. Some branching points are not well supported in the displayed tree, such as the lineages of ‘*Candidatus* Caldiarchaeum’ and ‘*Candidatus* Korarchaeum’. The former was affiliated rather with *Thaumarchaeota* in more comprehensive phylogenetic calculations (see e.g. Eme *et al.*, 2013).

Initial phylogenetic analyses of 16S RNA gene sequences from environmental studies placed the ‘mesophilic archaea’ (i.e. *Thaumarchaeota*), closely related to strain EN76^T^, as a deep-branching group of the *Crenarchaeota* (DeLong, 1992; Fuhrman *et al.*, 1992). However, later analyses based on full rRNA gene sequences, concatenated ribosomal protein sequences and full-genome sequence comparisons provided strong evidence that these mesophilic and aerobic AOA represent a distinct phylum, the *Thaumarchaeota* (Brochier-Armanet *et al.*, 2008; Spang *et al.*, 2010). Members of this phylum encode a specific set of information-processing genes that is distinct from those in the phyla *Crenarchaeota* and *Euryarchaeota*, as well as in the proposed phylum ‘*Korarchaeota*’ (Brochier-Armanet *et al.*, 2008; Spang *et al.*, 2010) and in other currently proposed phyla (Brochier-Armanet *et al.*, 2011; Rinke *et al.*, 2013; Spang *et al.*, 2013). Strain EN76^T^ is affiliated with group I.1b of the *Thaumarchaeota* (also known as the ‘soil group’) based on 16S rRNA gene phylogeny (Fig. 5), also showing a consistent phylogenetic clustering based on concatenated AmoAB protein sequences (Tourna *et al.*, 2011). ‘*Candidatus* Nitrososphaera gargensis’ Ga9.2 shares 97 % 16S rRNA gene sequence identity with strain EN76^T^ (Tourna *et al.*, 2011). ‘*Candidatus* Nitrosopumilus maritimus’ SCM1 is currently the only described pure culture of the second major group within group I.1a of the *Thaumarchaeota* (Könneke *et al.*, 2005; Walker *et al.*, 2010), and shares 85 % 16S rRNA gene sequence identity with strain EN76^T^. However, the name ‘*Nitrosopumilus maritimus*’ has not been validly published and does not have standing in nomenclature. Based on 16S rRNA gene sequence identity, *Thermofilum pendens* Hrk 5 (81 % 16S rRNA gene sequence identity) and *Methanothermus fervidus* DSM 2088^T^ (79 %) represent the closest related cultivated strains of species with validly published names from the phyla *Crenarchaeota* and *Euryarchaeota*, respectively (Fig. 5). Strain EN76^T^ has a DNA base composition of 52.7 mol% G+C (Tourna *et al.*, 2011), which is similar to that of ‘*Candidatus* Nitrososphaera gargensis’ Ga9.2 (48.4 mol%) and higher than that of group I.1a strains such as ‘*Candidatus* Nitrosopumilus maritimus’ SCM1 (34.2 mol%), ‘*Candidatus* Nitrosoarchaeum koreensis’ MY1 (32.7 mol%) and ‘*Candidatus* Nitrosoarchaeum limnia’ SFB1 (32.4 mol%) (Blainey *et al.*, 2011; Kim *et al.*, 2011; Spang *et al.*, 2012; Walker *et al.*, 2010). In conclusion, groups I.1a and I.1b differ greatly in their G+C content and form two highly supported distinct phylogenetic lineages based on both 16S rRNA and *amoA* gene sequences (Fig. 5).

### Description of *Nitrososphaera* gen. nov.

*Nitrososphaera* (Ni.tro.so.sphae′ra. N.L. adj. *nitrosus* full of natron; here intended to mean nitrous; L. fem. n. *sphaera* a ball, sphere; N.L. fem. n. *Nitrososphaera* the sphere producing nitrite).

Mesophilic to moderately thermophilic, acidophilic to neutrophilic, aerobic, autotrophic or mixotrophic, ammonia-oxidizing organisms. Cells are irregular coccoid. The major lipid is crenarchaeol and its regioisomer. The type species is *Nitrososphaera viennensis*.

### Description of *Nitrososphaera viennensis* sp. nov.

*Nitrososphaera viennensis* (vi.en.nen′sis. N.L. fem. adj. *viennensis* from Vienna, where the type strain was isolated and characterized).

Irregular cocci with a diameter of 0.78±0.13 µm. Occur as single cells and as clusters of several cells. Cells exhibit archaella (12.0±1.8 nm) and archaeal pili (6.4±1.3 nm) as cell appendages, and clearly discernible areas of high and low electron density and tubule-like structures in the cytoplasm. Cells possess an S-layer with p3-symmetry. Grows at pH 6–8.5, with an optimum at pH 7.5. The temperature optimum is 42 °C; grows at 28–47 °C. Energy is produced by oxidizing ammonia to nitrite with oxygen as electron acceptor. Optimal NH_4_Cl concentration for growth is 2.6 mM, but concentrations up to 15 mM are tolerated. Nitrite concentrations up to 10 mM are tolerated. Urea can be used as substrate. N_2_O is formed as a side product during ammonia oxidation. Mixotrophic growth is observed with bicarbonate and small carboxylic acids, i.e. pyruvate, α-ketoglutarate, oxaloacetate or glyoxylate, as carbon sources. The following substrates have a negative effect or inhibit growth under the conditions tested: ribose, glycerol, peptone, yeast extract, l-alanine, d-alanine, l-glutamine, l-aspartic acid, an amino acid mixture, Casamino acids, methylamine, trimethylamine, ethanolamine, methanolamine, nitrate, pyrimidine and purine.

The type strain, EN76^T^ ( = DSM 26422^T^ = JMC 19564^T^), was isolated from a garden soil in Vienna, Austria. The DNA base composition of the type strain is 52.7 mol% G+C.

### Description of *Nitrososphaeraceae* fam. nov.

*Nitrososphaeraceae* (Ni.tro.so.sphae.ra′ce.ae. N.L. fem. n. *Nitrososphaera* type genus of the family; L. suff. -*aceae* ending to denote a family; N.L. fem. pl. n. *Nitrososphaeraceae* the family of the genus *Nitrososphaera*).

The description is the same as for the genus *Nitrososphaera*. The type genus is *Nitrososphaera*.

### Description of *Nitrososphaerales* ord. nov.

*Nitrososphaerales* (Ni.tro.so.sphae.ra′les. N.L. fem. n. *Nitrososphaera* type genus of the order; N.L. suff. -*ales* ending to denote an order, N.L. fem. pl. n. *Nitrososphaerales* the order of the genus *Nitrososphaera*).

The name *Nitrososphaerales* refers to the former group I.1b (or ‘soil group’) within the phylum *Thaumarchaeota*. Cultivated organisms of this order have an irregular coccoid cell shape and occur predominantly in terrestrial ecosystems. By contrast, cells of all known organisms affiliated with group I.1a (order ‘*Nitrosopumilales*’) are rod-shaped. The order *Nitrososphaerales* comprises a highly supported distinct phylogenetic group based on 16S rRNA gene phylogeny (Fig. 5). The 16S rRNA genes of all members share ≥90 % sequence identity, as do all members of other phylogenetically well-defined groups, e.g. the tentative orders ‘*Nitrosopumilales*’ (represented by ‘*Candidatus* Nitrosopumilus maritimus’ SCM1; Könneke *et al.*, 2005), ‘*Nitrosotaleales*’ (represented by ‘*Candidatus* Nitrosotalea devanaterra’ Nd1; Lehtovirta-Morley *et al.*, 2011) and ‘*Nitrosocaldales*’ (represented by ‘*Candidatus* Nitrosocaldus yellowstonii’ HL72; de la Torre *et al.*, 2008). The names of these orders are currently not validly published, given the lack of representative organisms in pure culture, or depositions in culture collections. The type genus is *Nitrososphaera*.

### Description of *Nitrososphaeria* classis nov.

*Nitrososphaeria* (Ni.tro.so.sphae′ri.a. N.L. fem. n. *Nitrososphaera* the type genus of the type order of the class; N.L. suff. -*ia* ending to denote a class, N.L. neut. pl. n. *Nitrososphaeria* the class of the order *Nitrososphaerales*).

Cultivated strains within this class possess genes of both FtsZ- and Cdv-based cell division systems and have a topoisomerase IB. Similar to euryarchaeal strains, but in contrast to crenarchaeal strains, they have DNA polymerases B and D, eukaryote-like histones (H3/H4) and only one copy of the proliferating cell nuclear antigen and lack genes for RNA polymerase G (Brochier-Armanet *et al.*, 2011; Spang *et al.*, 2010). Crenarchaeol is the major core lipid and is not known to occur in any other bacterial or archaeal phylum (Pitcher *et al.*, 2010; Schouten *et al.*, 2008; Sinninghe Damsté *et al.*, 2002, 2012). Additionally, genes encoding an ammonia monooxygenase have been found exclusively in all lineages within the class, among all archaeal taxa described, and might therefore be considered a distinctive feature. So far, all investigated genomes of members of this class contain genes encoding key enzymes of the 3-hydroxypropionate/4-hydroxybutyrate pathway, including acetyl-CoA carboxylase, 4-hydroxybutyryl-CoA dehydratase and methylmalonyl-CoA mutase, suggesting that members of the phylum *Thaumarchaeota* might assimilate their cellular carbon via a modified version of this pathway (Berg *et al.*, 2007; Blainey *et al.*, 2011; Kim *et al.*, 2011; Mosier *et al.*, 2012a, b; Park *et al.*, 2012; Spang *et al.*, 2012; Walker *et al.*, 2010). The class comprises a highly supported monophyletic lineage in the 16S rRNA gene phylogeny of the *Archaea* (Fig. 5). The type order is *Nitrososphaerales*.
